# Classifying outcomes in secondary and tertiary care clinical quality registries—an organizational case study with the COMET taxonomy

**DOI:** 10.1186/s12913-022-08132-w

**Published:** 2022-06-21

**Authors:** Antero Vanhala, Anna-Rosa Lehto, Anu Maksimow, Paulus Torkki, Sanna-Maria Kivivuori

**Affiliations:** 1 Department of Public Health, Faculty of Medicine, University of Helsinki, P.O. Box 20, 00014 Helsinki, Finland; 2 Department of Information Service and Management, Aalto University School of Business, Espoo, Finland; 3 HUS Helsinki University Hospital, P.O. Box 100, 00029 HUS Helsinki, Finland

**Keywords:** Patient registry, Outcome assessment, Outcome measures, COMET, Comparative effectiveness research, Real-world data

## Abstract

**Background:**

The choice of what patient outcomes are included in clinical quality registries is crucial for comparable and relevant data collection. Ideally, a uniform outcome framework could be used to classify the outcomes included in registries, steer the development of outcome measurement, and ultimately enable better patient care through benchmarking and registry research. The aim of this study was to compare clinical quality registry outcomes against the COMET taxonomy to assess its suitability in the registry context.

**Methods:**

We conducted an organizational case study that included outcomes from 63 somatic clinical quality registries in use at HUS Helsinki University Hospital, Finland. Outcomes were extracted and classified according to the COMET taxonomy and the suitability of the taxonomy was assessed.

**Results:**

HUS clinical quality registries showed great variation in outcome domains and in number of measures. Physiological outcomes were present in 98%, resource use in all, and functioning domains in 62% of the registries. Patient-reported outcome measures were found in 48% of the registries.

**Conclusions:**

The COMET taxonomy was found to be mostly suitable for classifying the choice of outcomes in clinical quality registries, but improvements are suggested. HUS Helsinki University Hospital clinical quality registries exist at different maturity levels, showing room for improvement in life impact outcomes and in outcome prioritization. This article offers an example of classifying the choice of outcomes included in clinical quality registries and a comparison point for other registry evaluators.

**Supplementary Information:**

The online version contains supplementary material available at 10.1186/s12913-022-08132-w.

## Background

Clinical quality registries are increasingly common for tracking and investigating healthcare quality. The choice of outcomes is of paramount importance for capturing relevant and comparable information on the quality and effectiveness of care, and outcome harmonization is vital for improving patient results through activities such as organizational benchmarking and registry-based research. In this work, we set out to compare outcomes from clinical quality registries against a general, non-disease-specific outcome framework in a secondary and tertiary care academic hospital setting.

Outcomes are broadly defined as measurements or observations used to capture and assess the effect of treatment [1] and what people care about most when seeking treatment [2, 3]. In the context of clinical registries, outcomes are the recorded results of care.

Clinical quality registries record information about patients, their health status, and the healthcare received, typically focusing on patients with similar needs, medical conditions, or use of healthcare services [4]. Healthcare professionals, hospital managers, and other decision makers use registry data to monitor and assess healthcare outcomes (i.e., how different patients respond to different treatments or interventions), thus enabling evaluation of the quality and effectiveness of care [4, 5] and the improvement of patient care [6]. Benchmarking is a common and effective method for measuring, analyzing, and ultimately improving organizations’ performance by comparing the data on activities and results between similar organizations, including best-practice facilities [7]. Harmonization of outcome measurement is important for benchmarking and necessary to unleash the potential of clinical quality registries [6, 8, 9]. In quality registries, the benchmarking might entail the maturity of the registries themselves and ultimately the results of care recorded in the registries. Registries also hold a great untapped potential for real-world research [10–12]. Ideally, the framework for outcome choices would serve these different purposes.

Previously, there have been some efforts to harmonize the choice of outcome measures in clinical registries. The Outcome Measure Framework (OMF) of the American Agency for Healthcare Research and Quality (AHRQ) was created for registry development purposes [13–15]. In Sweden—a forerunner in clinical quality registries—patient-reported outcome measures (PROMs) in registries have been analyzed at least twice [16, 17], and the International Consortium of Health Outcome Measurement (ICHOM) has developed disease-specific outcome measurement standard sets [18, 19] to steer the choice of outcome measures. To date, however, the ICHOM standard sets do not seem to use any general outcome framework. Some health system authorities have developed outcome frameworks for reporting and monitoring purposes (e.g., NHS [20]) and others have developed frameworks for evaluating clinical registry maturity, capability, data quality, or design [21–25]. These frameworks are not, however, intended for classifying the choice of outcomes in registries.

For clinical trials, harmonization efforts of outcome measurement have taken strides forward, especially with core outcome sets (COS) that have been created for numerous clinical fields [26].

The COMET taxonomy, created by Dodd and others [27], is an outcome framework intended for developing and assessing COS in clinical trials and is based on previous conceptual and empirical work [28]. In a preliminary phase of this study, we performed a literature review in which we identified a total of 23 outcome frameworks that could be used in classifying choices of outcomes (see Additional file 1) and chose the COMET taxonomy for this study. In the deliberations, the following advantages were valued by the research team and hospital management in the COMET taxonomy: It classifies outcomes relevant to patients, including physiological outcomes and patient impact [18]; it is aligned with outcome unification efforts in the clinical trial setting [29, 30]; it is sufficiently granular without compromising comprehensibility (38 outcome domains classified into 5 core areas); it has instructions on classifying outcome measurement instruments to ensure consistency [31]; and it includes categories for resource use, which was seen as relevant for managerial and cost-effectiveness assessment purposes.

Use of the COMET taxonomy in the clinical quality registry context could offer a possibility to bring outcome measurement in clinical trials and registries a step closer, thus enabling broader registry research, comparability of findings, and better translation of clinical research results to the real-life context.

The aim of this study is to compare existing clinical registry outcomes against the COMET taxonomy and to assess the framework’s suitability in the clinical registry setting.

The goals of this research are as follows:

1. to classify the outcomes in HUS Helsinki University Hospital somatic clinical quality registries with the COMET taxonomy, and
2. to assess the suitability of the COMET taxonomy in classifying the choice of outcomes in real-world clinical quality registries.

Additionally, the work describes a practical example of how to carry out such a classification effort and provides a benchmark for other clinical quality registry evaluators.

## Methods

First, criteria for suitability of the COMET framework in classifying outcomes were discussed and agreed on within HUS Helsinki University Hospital quality management and the research team: The framework should 1) be feasible, meaning that the classification effort could be carried out in reasonable time and with reasonable resources; 2) have the ability to distinguish development needs in registries, meaning that the results can point to shortcomings in outcomes and differentiate between registries’ development stages; and 3) enable the classification of each outcome measure as unambiguously as possible. These criteria will be discussed in the Discussion -section of this article.

Second, we conducted an organizational case study with HUS Helsinki University Hospital (later HUS) clinical quality registries. Research data consisted of clinical outcome fields gathered from HUS somatic clinical quality registries. A total of 63 medical condition- or healthcare service (i.e., treatment)-specific [4] somatic registries were included in the study (Table 2).

HUS is a secondary and tertiary care academic hospital with 27,000 employees that serves a population of 2.2 million in Southern Finland. Certain disease entities have been centralized to HUS nationally (total population of 5.5 million). Annually, around 680,000 individual patients (2.7 million visits) are treated at HUS [32]. HUS has deployed clinical quality registries for clinical use, quality, effectiveness, and research purposes. Teams of expert clinicians have chosen the outcome measures, thus representing a local (or, in some registries, a national) expert consensus. The outcomes are recorded in a structured format, ensuring high measurement consistency. The broadness across disease areas combined with accessibility make the HUS quality registries an excellent case study target for the validation of outcome frameworks.

All data entry field titles were extracted from registry interfaces (59 registries) or technical definition documents (4 registries) and gathered in a separate research table. The title of each data entry field corresponds to the name of the variable. We also extracted items from the reporting functionalities, if functional. For each data entry field, we recorded data category, data subcategory, data entry field title, and input unit (e.g., kg, cm) or input choices (drop-down list or open text). No personal data were collected. Furthermore, for each registry, we recorded the following: the number of patient entries, reporting functionality (yes or no), and patient questionnaire functionality (yes or no).

Third, each input field was assessed with the following process: 1) identify whether the item is a potential outcome measure; 2) if yes, classify the item into the corresponding outcome domain(s) within the COMET taxonomy. Additionally, we chose to characterize outcome measurement instruments in our data with the following methods found in the literature:

- Measurement method: Physiological measure (e.g., blood sugar)/professional-reported measure (contains a significant degree of subjectivity, e.g., Eastern Cooperative Oncology Group Performance Status Scale, ECOG)/PROM (e.g., EQ-5D)/patient-reported experience measure (PREM, e.g., patient evaluation of communication quality) (adapted from [33, 34]).

For standardized patient-reported questionnaire instruments:

- Scope: General (e.g., EQ-5D)/disease-specific (e.g., Oxford Hip Score) [35]
- Dimensionality: Composite (e.g., EORTC QLQ-C30)/unidimensional (e.g., unique question of global quality of life) [35]

All methods were carried out in accordance with relevant guidelines and regulations. The study was reviewed and approved by the Research Administration of the Helsinki and Uusimaa Hospital District (Research Director resolution, 20 February 2020), and the data security plan and measures were implemented in accordance with relevant guidelines.

## Results

### Classification of outcomes in HUS clinical quality registries using the COMET taxonomy

In total, the 63 clinical quality registries contained 23,833 data fields, 9493 of which were identified as potential outcome measures. The median (range) number of outcomes per clinical registry was 118 (15 to 751). The number (and %) of registries containing each COMET domain with the median (range) number of items per registry is presented in Table 1. Some outcome measures in 21 registries were marked “national” or “cancer information notification” (related to Finnish national cancer registries [36]), but mostly, we did not find prioritization of outcome measures.

**Table 1:** Frequency of measures in each COMET outcome domain in investigated registries

Core area	Outcome domain	At least once, nr of registries (%)	Median number of outcome measures per registry (range)
Death	1. Mortality/survival	35 (56)	6 (1 to 49)
Physiological/clinical	*Any physiological/clinical domain 2–24*	*62 (98)*	*47 (4 to 427)*
	2. Blood and lymphatic system outcomes	18 (29)	1 (1 to 5)
	3. Cardiac outcomes	28 (44)	2.5 (1 to 151)
	4. Congenital, familial, and genetic outcomes	4 (6)	1 (1 to 1)
	5. Endocrine outcomes	8 (13)	2 (1 to 7)
	6. Ear and labyrinth outcomes	8 (13)	1 (1 to 6)
	7. Eye outcomes	14 (22)	2 (1 to 87)
	8. Gastrointestinal outcomes	27 (43)	3 (1 to 54)
	9. General outcomes	48 (76)	5 (1 to 46)
	10. Hepatobiliary outcomes	13 (21)	2 (1 to 46)
	11. Immune system outcomes	19 (30)	2 (1 to 28)
	12. Infection and infestation outcomes	30 (48)	2 (1 to 23)
	13. Injury and poisoning outcomes	9 (14)	1 (1 to 6)
	14. Metabolism and nutrition outcomes	12 (19)	1 (1 to 40)
	15. Musculoskeletal and connective tissue outcomes	25 (40)	3 (1 to 134)
	16. Outcomes relating to neoplasms: benign, malignant, and unspecified	25 (40)	13 (1 to 34)
	17. Nervous system outcomes	33 (52)	2 (1 to 126)
	18. Pregnancy, puerperium, and perinatal outcomes	7 (11)	1 (1 to 2)
	19. Renal and urinary outcomes	25 (40)	4 (1 to 64)
	20. Reproductive system and breast outcomes	11 (17)	4 (1 to 31)
	21. Psychiatric outcomes	10 (16)	4.5 (1 to 29)
	22. Respiratory, thoracic, and mediastinal outcomes	25 (40)	3 (1 to 234)
	23. Skin and subcutaneous tissue outcomes	30 (48)	1,5 (1 to 45)
	24. Vascular outcomes	39 (62)	5 (1 to 34)
Life impact	*Any life impact domain 25–33*	*58 (92)*	*13 (4 to 361)*
	*Any functioning domain 25–29*	*39 (62)*	*23 (2 to 312)*
	25. Physical functioning	37 (59)	11 (1 to 239)
	26. Social functioning	23 (37)	3 (1 to 36)
	27. Role functioning	31 (49)	3 (1 to 16)
	28. Emotional functioning/well-being	22 (35)	7 (1 to 70)
	29. Cognitive functioning	22 (35)	3 (1 to 30)
	30. Global quality of life	3 (5)	1 (1 to 2)
	31. Perceived health status	22 (35)	4 (1 to 32)
	32. Delivery of care	53 (84)	4 (1 to 58)
	33. Personal circumstances	18 (29)	1 (1 to 28)
Resource use	*Any resource use domain 34–37*	*63 (100)*	*37 (5 to 233)*
	34. Economic	27 (43)	4 (1 to 73)
	35. Hospital	48 (76)	3.5 (1 to 14)
	36. Need for further intervention	63 (100)	35 (4 to 225)
	37. Societal/carer burden	9 (14)	2 (1 to 49)
Adverse events	38. Adverse events/effects	55 (87)	6 (1 to 30)

Number (and %) of registries containing at least one outcome measure pertaining to each COMET taxonomy outcome domain [27], and median (and range) number of outcome measure items in the registries containing that domain. Total number of registries was 63. Please note, as instructed by COMET authors, Domain 30. Global quality of life is reserved for unidimensional health-related quality of life questions, and composite scores for quality of life should be recorded for all domains that are included in the full questionnaire tool sub-questions; Domain 38. Adverse events/effect: Specified adverse effects of care are classified into their relevant physiological domains and marked with an additional sub-class, “Harm”; and Domain 38. Adverse events/effects is reserved for unspecified adverse events or effects (e.g., “Other adverse effect”)

The COMET taxonomy core areas identified in each registry are presented in Table 2.

**Table 2:** List of investigated clinical quality registries and occurrence of COMET taxonomy core areas

Clinical quality registry	COMET core areas found					Clinical quality registry	COMET core areas found				
	Death	Physiological/clinical	Life impact	Resource use	Adverse events		Death	Physiological/clinical	Life impact	Resource use	Adverse events
Asthma	○	●	●	●	●	Multiple sclerosis	○	●	●	●	●
Back surgery	○	●	●	●	●	Nasal airway surgery	○	●	●	●	●
Bariatric surgery	○	●	●	●	●	Nephrology (N)	●	●	●	●	●
Brain tumors (N)	●	●	●	●	●	Neuromodulator	○	●	●	●	●
Breast cancer - Oncology	●	●	⋄	●	●	Pacemaker	●	●	–	●	●
Breast cancer - Surgery (N)	●	●	●	●	●	Pain	○	●	⋄	●	●
Cataract	○	●	●	●	●	Pediatric and adolescents’ cancer	○	●	●	●	●
Cerebral stroke (N)	○	●	●	●	●	Pediatric back surgery	○	●	●	●	●
Cleft Palate and Craniofacial	○	●	●	●	●	Pediatric cancer	●	●	●	●	●
Colon and rectal cancer - Oncology	●	●	⋄	●	●	Pediatric fractures	○	●	●	●	●
Colon and rectal cancer - Surgery	●	●	●	●	●	Pediatric heart surgery	●	●	●	●	●
COVID19	●	●	●	●	–	Prostate cancer - Oncology	●	●	⋄	●	●
Diabetes (N)	○	●	●	●	●	Prostate cancer - Surgery	○	●	●	●	●
Epilepsy	○	●	●	●	●	Rare diseases (N)	●	–	⋄	●	–
Fractures	○	●	●	●	–	Renal cancer - Oncology	●	●	⋄	●	●
Gynecologic cancer (N)	●	●	●	●	●	Renal cancer - Surgery (N)	○	●	⋄	●	●
Head and neck cancer - Oncology	●	●	⋄	●	●	Retina	○	●	⋄	●	●
Head and neck cancer - Surgery (N)	●	●	●	●	●	Rheumatism	●	●	●	●	●
Heart and lung transplant	●	●	●	●	●	Sarcoma and GIST	●	●	⋄	●	–
Heart surgery	●	●	–	●	●	Skin cancer - Oncology (N)	●	●	⋄	●	●
Hepatitis C (N)	○	●	⋄	●	–	Skin cancer - Surgery (N)	●	●	–	●	●
Hernias	○	●	●	●	●	Spinal injury	○	●	●	●	●
Hip surgery (N)	○	●	●	●	●	Tissue flap surgery (N)	●	●	–	●	●
Inflammatory bowel disease	○	●	●	●	●	Transcatheter valve replacement	●	●	●	●	●
Intensive care	●	●	●	●	●	Trauma and injury (N)	●	●	⋄	●	–
Invasive cardiology	●	●	⋄	●	●	Upper gastroenterological cancer	●	●	⋄	●	●
Kidney and pancreas transplant	●	●	●	●	●	Urogynecology (N)	○	●	●	●	●
Knee surgery (N)	○	●	●	●	●	Urothelial cancer (N)	●	●	●	●	●
Liver transplant	●	●	●	●	●	Vascular anomalies	○	●	●	●	●
Lung cancer (N)	●	●	●	●	●	Vascular surgery	●	●	⋄	●	●
Lymphoma - Oncology	●	●	⋄	●	●	Venous surgery (N)	○	●	–	●	–
Medical Emergency Team, Resuscitation	●	●	⋄	●	–						

Explanations: ● Core area was explicitly found in the registry, − Core area was not found in the registry, ○ Death outcome was not explicitly tracked, but status can be accessed any time, ⋄ No other life impact domain was found except for Domain 32 Delivery of care that includes, for example, waiting times. (N) The registry contained nationally tracked measures

A death core area was found in over half of the registries. Survival measures were common in cancer and surgery-related registries. However, thanks to a data integration with the Finnish population registry, it is possible to view an individual patient’s living status in all registries.

Physiological/clinical core area outcomes were observed in almost all registries and represented the largest number of all outcome items both overall and in most registries individually. Domain 9 (General outcomes) was most commonly found (76%). The occurrences of most physiological/clinical outcome domains reflected the registry’s clinical area.

Life impact core area outcomes were found in nearly all registries. However, functioning domains (25–29) were present in only two-thirds of registries, physical functioning (domain 25) being the most common, and emotional functioning/well-being (domain 28) and cognitive functioning (domain 29) being the least common. Domain 30 (Global quality of life) was found in only three registries and always as part of a questionnaire instrument. Outcome Domain 32 (Delivery of care) contains measures of waiting time and treatment delivery in addition to patient experience measures and was found in 84% of registries. Should the delivery of care domain (32) be omitted from the analysis, 63% of the registries contained some other life impact domain (see Table 2 for details).

Resource use was measured in all quality registries. The level of detail for measuring resource use varied greatly. Detailed care-related information (e.g., medication dosage or implant data) inflated the number of items in some registries.

The adverse events domain (38) is reserved for previously undefined or other broadly labelled items that implicate harm or unintended effect of care. Otherwise, adverse events were classified into the corresponding outcome domain with an additional label for harm. Most registries contained a measure or field for non-specified adverse effects or events, usually combined with a free text field. Several registries contained a SNOMED-based adverse event recording and are thus classified into their corresponding COMET taxonomy domains.

### Other characterizations of outcome measurement instruments

We also characterized the measurement method for each outcome. PROMs were of special interest. Overall, 1845 patient-reported items were identified. Thirty registries (48%) contained some PROMs. The median number of patient-reported items in these registries was 38.5 (ranging from 2 to 301). One or more generic instruments of health-related quality of life were found in 18 registries (29%), the most common being the 15D Health-related quality-of-life questionnaire (17 registries), followed by single occurrences of EQ-5D and WHOQOL-BREF questionnaires. Patient-reported disease- or symptom-specific questionnaires were found in 23 registries (37%) with a great variety of different instruments. Both standardized and unstandardized questionnaire tools were identified. Patient-reported experience measures (PREMs) are questionnaires that gather patients’ subjective experiences while receiving care [37]. Ten registries contained some PREMs, with a median (range) of 4.5 (1 to 14) items. PREMs were mostly unstandardized questions regarding general experience of care, satisfaction with care received, or experiences with different aspects of the service (e.g., shared decision-making, information clarity, feelings of safety). Physician-reported items were relatively rare. Physiological measures were the most abundant, as expected.

## Discussion

### Choices of outcomes included in HUS clinical quality registries

Our findings from the HUS clinical quality registries showed great variation in outcome choices and measurement maturity between registries. Overall, the number of outcome measures per registry was found to be very high compared with common COS in clinical trials [27] and ICHOM standard sets [19]. Furthermore, prioritization of the most important outcomes was not usually visible in registry user interfaces. We observed some overlap of outcomes inside registries.

The occurrences of COMET taxonomy outcome domains varied markedly between registries. Life impact and death core areas showed the most room for development. Arguably, survival outcomes are not pertinent in some diseases, but we believe they should be explicitly tracked for at least the purposes of overall hospital mortality rate in secondary and tertiary care hospitals. In the HUS clinical quality registries, it is, however, possible that survival is tracked outside the registries as such data are readily available from the hospital electronic health record. The life impact core area showed clear implications for improvement: Excluding Domain 32 (Delivery of care), which mostly contained administrative or process-oriented measures, the life impact core area seemed to have few occurrences of outcome items. Patient-centric measures, mostly found in the life impact core area in the COMET taxonomy, tend to be associated with more mature registries [21], and our findings thus indicate low- to middle-level maturity. We recommend adopting outcomes from life impact domains with patient-reported instruments; for example, the global quality of life question (Domain 30) could be adopted relatively easily in all registries. Of the other core areas, resource use domains were found in all registries and physiological/clinical domains in all but one (which tracked diagnosis rather than outcomes). The variation in domains and number of items seems to be somewhat explained by the registry’s disease area and medical specialty.

Similar findings have been reported previously in US and Swedish clinical registries, leading to harmonization efforts [14, 16]. Although hospital-level registries might allow for a larger number of tracked outcomes than their national and international counterparts, we recommend defining minimum sets based on clinical trial COS, such as from the COMET initiative [38], and real-world outcome sets, such as ICHOM standard sets [19], to help guide the data gathering.

PROMs in HUS clinical quality registries were less common than in Swedish national quality registries (48% versus 86% in Sweden) [17]. The findings on generic health-related quality of life instruments were similar: 29% in HUS registries versus 35–40% in the Swedish registries [17]. However, the choice of measure was not the same: 15D—a generic measure developed in Finland [39, 40]—was most used in HUS registries, whereas other countries most often used EQ-5D and SF-36/RAND-36 [41–45]. The choice of PROMs should be harmonized with international development to enable broader comparability and research collaboration.

### COMET taxonomy for classifying outcome choices in clinical quality registries

To our knowledge, the COMET taxonomy has not been used previously to classify outcome choices in clinical quality registries. This research offers an example of conducting an analysis of clinical registry outcome choices.

Overall, the COMET taxonomy was found mostly suitable and useful for the purpose. Although it provided actionable knowledge on the current state of clinical registries with a reasonable effort, we identified several suggestions for improvement. We will discuss the suitability of the COMET framework point by point.

#### Feasibility

The time and resources needed to carry out the data gathering and analysis proved to be feasible. The classification effort was carried out by one researcher within a period of 2 months of full-time work for almost 24,000 lines of data. Because this kind of classification only needs to be done once and the subsequent changes in outcome measures only require the classification of the new items, we assess that for time and resource use, the COMET taxonomy is feasible. There is some learning curve to accurately and consistently classify similar items, and we suggest that the classification should be done by a designated expert with medical training, preferably by specialists in each field.

#### Ability to differentiate development needs in registries

We identified core area- and outcome domain-level differences in outcome measurement between registries. The additional characterizations of measurement method proved valuable in assessing where patient-reported data were gathered. Although not all registries need to include all domains, we believe that the COMET taxonomy offers a reasonable guide to systematically develop outcome measurement inside registries and for managers to identify registries that need improvement focus. The potential to identify meaningful differences in outcome measurement also encourages the use of the taxonomy for benchmarking quality registry maturity.

#### Unambiguous classification of each outcome item

The ease and achievability of discrete classification of each item is a desirable attribute for a framework. The possibility to categorize items in multiple domains simultaneously is a feature of COMET taxonomy, although ideally, each item would fall clearly into one category. In this regard, the COMET taxonomy fared well in most core areas, but we found room for improvement, especially in the resource use core area and the global quality of life domain (30), which we will discuss below. All outcomes could be classified into some domain. In general, the instructions provided on the COMET initiative website offered guidance, but we hope to see more exhaustive instructions in the future.

The definitions of outcome domains in the COMET core area resource use posed some classification challenges. The main difference between resource use in clinical trials and real-world clinical settings arises from defining the intervention. Whereas clinical trials are designed to have a clear intervention, the interventions in clinical quality registries tend to be defined loosely and include many changing components. In this research, we classified all interventional elements—including the registry’s primary intervention—into outcome Domain 36 (Need for further intervention), while identifying the shortcomings of this approach. Other resource use elements were classified into their corresponding domains. Furthermore, personnel resource use (e.g., consultations) was classified into Domain 35 (Hospital) and outsourced services into Domain 34 (Economic). We would be happy to see even clearer definitions in this core area to aid in similar classification efforts. Additionally, we believe there is a need to subdivide the resource use domains to appreciate the use of different monetary and nonmonetary resources for managerial purposes and to enable future registry-based cost-effectiveness research.

In the life impact core area, Domain 30 (Global quality of life) in the current taxonomy is reserved for unidimensional quality of life questions. As Dodd [27] and Macefield [46] proposed, the composite indices are classified into all relevant domains based on individual items. However, the composite scores of quality of life (e.g., EORTC QLQ-C30) are very relevant for managerial, health economic, and comparison purposes, and thus, we suggest the addition of subcategories—30a (Global quality of life, unidimensional index) and 30b (Global quality of life, composite index)—which would capture the composite score. We propose that the subdomain 30b would include the composite scores from validated general or disease-specific health-related quality of life instruments.

### Other characterizations of outcome measurement instruments

Apart from the COMET taxonomy, we included some characterizations of outcome measurement instruments in our analysis: measurement method, scope, and dimensionality (for patient-reported instruments). The COMET taxonomy is not intended to classify measurement method, but drawing from our study, we recommend other registry evaluators to record the measurement method of each outcome field using “patient-reported,” “professional-reported,” and “physiological (objective)” categorization to enable better benchmarking of outcome measures against other quality registries, as in our comparison with Swedish clinical registries. With measurement method, we also found that it is possible to map the COMET taxonomy domains to the OMF [14] outcome categories with reasonable accuracy (but not vice versa) to enable further comparability.

### Limitations of this study

The main limitation of this study is related to the possible variation in classifying outcome items: At times, an outcome item might be interpreted to belong to more than one domain. For example, the patient-reported experience of pain could be classified into either a physiological domain or a life impact domain. We used the COMET taxonomy classification instructions [31] throughout the classifying effort and listed outcome items that were found to be ambiguous to ensure consistent classification across registries. The COMET authors suggest that when in doubt, the item should be classified into all possible domains. Consequently, the registries might seem to include more outcome domains than they actually do. Medical directories and information sources were used to help understand the outcome items in their clinical context. Considering the size of the data set, the conclusions should remain essentially the same despite the possible inaccuracies. Variation could be further decreased with the use of specialists in each field and by detailing the COMET instructions. Another limitation of the study is the inclusion of registries from only one hospital, which could affect the generalizability of our findings regarding the COMET taxonomy.

## Conclusions

In conclusion, we found the COMET taxonomy to be mostly suitable and useful in a clinical quality registry context, and with some reservations, we would recommend its use for clinical registry developers, researchers, and hospital managers to assess outcome measurement and to guide the choice of outcomes. Our main concerns relate to the ambiguity of certain domains of the framework, which should be considered in similar classifying efforts and in future development of the COMET taxonomy and its guidance. Use of the COMET taxonomy in conjunction with characterization of measurement method should be sufficient for benchmarking registry maturity and could bring us one step closer to efficient quality of care benchmarking between organizations [9]. We believe that there are benefits to sharing the same model between clinical trials and registries; it steers registry development and research, leads to more comparable and relevant clinical registry data, bridges the gap from trials to practice by helping understand clinical trial results in a local context, and encourages registry research that could combine data from multiple organizations.

Our research on HUS Helsinki University Hospital registries supports previous findings of variation in the choice of outcomes in clinical quality registries and the need for harmonization. There are very few published reviews that cover larger numbers of registries. More primary research on registries at the national and international levels is needed, as well as meta-analyses of existing narrower reviews. In our view, there is a clear need for a unified framework to elucidate the full picture of outcome choices.

## Supplementary Information

**Additional file 1.**

## Data Availability

The data that support the findings of this study are available from HUS Helsinki University Hospital and the Helsinki and Uusimaa Hospital district, but restrictions apply to the availability of these data, which were used under license for the current study and thus are not publicly available. Data are, however, available upon reasonable request by contacting the corresponding author.

## Abbreviations

AHRQ: American Agency for Healthcare Research and Quality
COS: Core outcome set
ECOG: Eastern Cooperative Oncology Group Performance Status Scale
HUS: HUS Helsinki University Hospital
ICHOM: International Consortium of Health Outcome Measurement
OMF: The Outcome Measure Framework
PROM: Patient-reported outcome measure
PREM: Patient-reported experience measure
