# Supplementary material for: Classifying outcomes in secondary and tertiary care clinical quality registries—an organizational case study with the COMET taxonomy
Source: BMC Health Serv Res. 2022 Jun 21;22:806. doi: 10.1186/s12913-022-08132-w (PMC9215071; doi:10.1186/s12913-022-08132-w)
Supplement: Supplementary file 1 — Additional file 1. [file 12913_2022_8132_MOESM1_ESM.docx]

# Supplementary material

# Description of Comprehensive literature review of Outcome Frameworks

# Purpose

This comprehensive literature review was conducted as a preparatory work for the main research. The goal of this review was to map suitable frameworks for classifying outcomes in clinical quality registries. This review is offered as a supplementary material to make available the list of possible frameworks to other researchers.

# Methodology

We conducted a comprehensive literature review from OVID Medline and Scopus databases. Search terms combined words ’outcome’ or ’outcomes’ to words ’taxonomy’, ’classification’, ’ontology’, ’categorisation’/’categorization’ or ’set’. The terms needed to occur no more than three words apart in article title, abstract, key words, subject heading, or other metadata. Searches were limited to published medical literature in English. Search results were screened for duplicates and analysed following PRISMA framework [A1]. Three screening rounds were performed: based on article title, based on article abstract, and based on full text. Main inclusion criterion was that publication needed to contain an original (or clearly further developed from another) outcome classification framework that was designed for general use (as opposed to disease-specific use). We included classification models intended for clinical trial, clinical registry, and clinical practice to allow a greater number of results. If a classification model had multiple versions, the latest published version was identified, retrieved (if necessary) and included in the review. Results of the literature searches were further complemented with snowballing approach.

The articles were assessed by one researcher (AV), and thus this preparatory work is a comprehensive literature review and not a full systematic review.

The selection of framework for the main study was based on previously identified criteria and deliberations during the analysis of review results. Predefined criteria were: the framework classifies outcomes relevant to patients including physiological and patient impact [A2], it is aligned with outcome unification efforts in clinical trial setting [A3, A4], and it includes categories for resource use which was seen relevant for managerial and cost-effectiveness assessment purposes. A pilot classification was done in one quality registry to test the practical usability of the most promising two frameworks. The pilot led to additional criteria: the framework is sufficiently granular without compromising comprehensibility and it offers clear instructions on classifying outcome measurement instruments to ensure consistency.

For managerial and practical purposes, we identified from the literature other possible ways of characterizing outcome measures in clinical quality registries. The primary purpose of these additional characterization methods was to increase the usability of the data for benchmarking, analytic, decision-making, and development purposes.

# Results

Total of 23 outcome classification frameworks were identified and reviewed: 9 from literature searches and 14 from other sources. PRISMA-flow diagram of the literature review presented in Figure A-1 Summary of outcome classification frameworks is presented in Table A-1. Most models fulfilled our criteria of measuring patient-centric outcomes. Only few offered sufficient granularity and comprehensibility for our purpose. Several models were intended for use in both trial and clinical practice setting, other models limited to trials or practice.

Two of the frameworks were seen as most promising when reviewing the results: Outcome Measure Framework (OMF) and COMET taxonomy and were tested in a pilot classification of outcomes from one quality registry.

**23 articles included**

5068 records identified through database searches (OVID Medline 1734 records and Scopus 3334 records)

4514 records after duplicates removed

4514 records screened based on title and abstract

43 full text articles assessed for eligibility

4471 records excluded

20 full text articles excluded:

-14 did not contain an original outcome framework

- 6 contained an outcome framework that has a later version that was retrieved

14 records identified through other sources

Identification

Screening

Eligibility

Included

Figure A-1 PRISMA-flow chart of the comprehensive literature review on outcome classification frameworks.

Table A-1 Results of the comprehensive literature review on outcome classification frameworks.

| Author(s) (Year) | Description | Reference |
| --- | --- | --- |
| WHO (1948) | World Health Organization's original definition of health as described in WHO constitution: physical, mental, and social. The division has been used as a framework itself and has influenced many later outcome frameworks. Intended as a general and universal description of health. | [A5] |
| Fries et al. (1980) | Originally developed conceptually for clinical practice and trials in arthritis but later applied to other illnesses. 5 D -model focus on harms that may result from treatment: Death, Disability, Discomfort, Drug toxicity, and Dollar cost, that are further divided to subcategories and subsubcategories. | [A6] |
| Tarlov et al. (1989) | The Medical Outcomes Study (MOS) framework was developed for the purposes of the study and encompassing both clinical practice and trial use. It contains three categories: Structure of Care, Process of Care, and Outcomes. These are further divided into subcategories, the Outcomes category containing four subcategories in total: clinical endpoints, functional status, general well-being, and satisfaction with care. Outcome subcategories are further divided into sub subcategories. | [A7] |
| Lang et al. (1990) | The authors classified outcome findings from previous literature into 15 emergent outcome categories. The resulting framework is intended for nursing practice and research purposes. | [A8] |
| Kozma et al. (1993) | The Economic, clinical, and humanistic outcomes model (ECHO) was originally developed to model outcomes of pharmaceutical treatments and services for real-world purposes. As the name suggests, outcomes are divided into economic (e.g., Costs), clinical (medical events, and humanistic (functional status or quality of life) | [A9] |
| Wilson and Cleary (1995) | Conceptually developed framework that has been cited frequently by later literature and is intended for usage in clinical practice and trials. The model contains five outcome categories: biological and functional variables, symptom status, functional status, general health perceptions, and overall quality of life. | [A10] |
| Nelson et al. (1996) | Researcher-developed outcome framework for practical quality improvement initiatives in real-world setting. The framework divides outcomes to functional, clinical, satisfaction, and cost outcomes, which are all divided into subcategories. | [A11] |
| Seymour et al. (1997) | The authors developed and outcome characterization system classifying outcome results from previous literature, especially clinical trials. The authors divide outcomes to mortality, clinical or other intermediate results, and final outcomes or quality of life. | [A12] |
| Jennings et al. (1999) | Emergent outcome classification from comprehensive literature review from 1974 onwards. Outcomes are divided into patient-focused, provider-focused, and organization-focused. The model is intended for real-world use and has subcategories. | [A13] |
| Pfeifle et al. (1999) | Based on Wilson & Cleary's (1995) model and adapted into educational objectives cognitive domains by Bloom (1956). The framework is intended for use in future research. The authors divide individual patient outcomes into factual-level outcomes, application-level outcomes, and problem-solving outcomes. These are further divided into subcategories. | [A14] |
| Evans et al. (2001) | Authors developed the outcome categorization based on previous practical work on national healthcare systems, and it was created quality improvement purposes. The categorization was originally focused on healthcare system -level quality assessment but is expanded to cover all system levels including clinical practice. | [A15] |
| Valderas and Alonso (2008) | Classification system for PRO measures in clinical practice and trials, based on Wilson & Cleary (1995) and WHO ICF. The framework has three axes: Construct, Measurement, and Population, of which the construct axis contains an outcome framework. The outcome domains are symptoms, functional status, health perceptions, health-related quality of life, and other health-related constructs. The model uses ICD-10 main categories for symptoms, and ICF subcategories for functional status. | [A16] |
| Wood et al. (2008) | A binomial categorization used by the authors to assess outcome bias in clinical trials. Outcomes are divided in subjective and objective outcomes, and further divided into subcategories. | [A17] |
| Porter (2010) | Porter's Outcome Measure Hierarchy was presented in a seminal piece on value in healthcare and is intended to real-world use. Outcomes are divided into health status achieved or retained, process of recovery (e.g., Time to recovery and disutility of care), and sustainability of health (freedom from recurrences and long-term effects). | [A18] |
| Davey et al. (2011) | An outcome framework developed by the authors for the purpose of assessing outcomes in clinical trials. Outcomes are divided into 23 categories. | [A19] |
| Smith et al. (2015) | An emergent outcome framework from a review of trials in Cochrane library that was created for the purposes of this research. Authors specify that they do not attempt to define an outcome domain system. The framework has 15 outcome categories. | [A20] |
| Drummond (2015) | Health economics framework that classifies outcomes into final and intermediate results of care. Final outcomes are divided into uni- and multidimensional, and disease-specific or general. It essentially characterizes measurement methods. | [A21] |
| WHO (2017) | WHO's International Classification of Functioning, Disability and Health (ICF) is an influential system for classifying a broad array of effects, health states and results. The system contains six taxonomic levels and is very detailed. | [A22] |
| Moorhead et al. (2018) | The Nursing Outcome Classification (NOC) was originally developed in the early 1990's and was based on the Medical Outcomes Study (MOS) by Tarlov et al. (1989). The current 6th edition is a detailed classification system of nursing-related outcomes with three taxonomic levels. The NOC is mainly used for clinical practice and nursing development. | [A23] |
| Leavy et al. (2019) | The Outcomes Measure Framework (OMF) was developed by the US Agency for Healthcare Research and Quality, and published by Gliklich et al. (2014), this article having the updated version. The OMF is intended for evaluating, harmonizing, and developing clinical registries and contains categories for all registry data: Characteristics (e.g., population), Treatment, and Outcomes. Outcomes are divided into survival, clinical response, events of interest, patient-reported, resource utilization, impact on non-participant, and experience of care. | [A24] |
| Dodd et al. (2018) | The COMET taxonomy was developed for evaluation and harmonizing of Core Outcome Sets for clinical trials as a part of the Core Outcome Measures in Effectiveness Trials (COMET) initiative. The taxonomy divides outcomes into 5 core areas: death, physiological/clinical, life impact, resource use, and adverse events, which are further divided into subcategories. | [A25] |
| Boers et al. (2019) | The OMERACT filter was originally developed for harmonizing outcome choices in rheumatism trials but has later been applied to many other clinical fields. The current version is 2.1. Outcomes in OMERACT filter are divided to manifestations/abnormalities, life impact, death/lifespan, and societal/resource use. | [A26] |
| NIH (2020) | PROMIS (Patient-Reported Outcome Measurement Information System) is an initiative that offers patient-centric sets of measurement tools. PROMIS-framework uses WHO's tripartite definition of health and divides patient-reported outcomes into physical, mental, and social, which are all further divided into more detailed domains. | [A27] |

# Conclusions

Outcome taxonomy (later 'COMET taxonomy') created by Dodd and others [A25] was chosen as the model for classifying outcome measures in this research. The taxonomy was created for clinical trial core outcome set development and assessment purposes. It is based on conceptually developing the previous work of Smith and others [A20] which in turn is based on emergent findings from reviewing Cochrane library reviews.

The COMET taxonomy and the Outcome Measures Framework (OMF) [A24] were piloted in one quality registry. The research teams made the remarks presented in Table A-2 based on framework documentation and experiences from the piloting.

Table A-2 Remarks on COMET taxonomy and Outcome Measures Framework (OMF) against selection criteria.

| ***Criterion*** | **COMET** | **OMF** |
| --- | --- | --- |
| Outcomes relevant to patients | Yes, includes physiological as well as patient life impact domains. | Yes, includes clinical response and patient-reported categories. However, we consider ‘patient-reported’ a measurement method rather than outcome category. |
| Aligned with clinical research | Yes, framework was created for clinical research setting. | No, framework was created for clinical registry setting. |
| Granular yet comprehensible | Yes, divided to two taxonomic levels that are clearly defined. Allows for analysis on upper core area level or lower domain level. | Somewhat, one well-defined taxonomic level. Subcategories listed but not defined. |
| Clear classification instructions for consistency | Yes, classification instructions are available at COMET website. | Somewhat, the OMF design and other documents contain some guidance but seems to limit to the upper taxonomic level. |
| Contains resource use | Yes, includes Resource use core area and domains. | Yes, includes Resource utilization category with subcategories. |

We believe that of the reviewed models COMET taxonomy fulfilled best our criteria: it classifies outcomes relevant to patients including physiological and patient impact [A1], it is aligned with outcome unification efforts in clinical trial setting [A2, A3], it is sufficiently granular without compromising comprehensibility (38 outcome domains classified into 5 core areas), it has instructions on classifying outcome measurement instruments to ensure consistency [A28], and it includes categories for resource use which was seen relevant for managerial and cost-effectiveness assessment purposes. Authors of COMET taxonomy has also called for 'feedback from researchers applying the taxonomy in their clinical settings to demonstrate further validation of the taxonomy or to highlight any necessary changes' [A25].

Another framework, the Outcome Measure Framework [A24, A29] has been implemented in clinical registries before and would have been an obvious choice but we assessed it to have the following drawbacks: with only one well-defined taxonomic level we could not fulfil the criterion of sufficient granularity, classification instructions were not as clear in COMET taxonomy, and we considered ‘Patient-reported’ -category to be a measurement method and not an outcome domain.

The choice of framework may also help in bridging the gap between clinical quality registry and clinical trial outcome measurement [A30] which favoured the selection of the COMET taxonomy.

The literature review has some limitations. We acknowledge that the process of selecting the framework was not fully systematic and was based on deliberations within the research team. This review was preliminary work and thus should not be seen as standalone research. Additionally, some articles may not have appeared in our searches because the keywords needed to occur maximum of three words apart to limit the number of search hits. The high proportion of articles identified from other sources may suggest that articles were left out from the original search. Furthermore, the choice of articles was based on one reviewer’s work which may cause bias.

**References**

[A1] Prisma. PRISMA, Preferred Reporting Items for Systematic Reviews and Meta-Analyses. 2015.

[A2] Porter M. What Is Value in Health Care? New England Journal of Medicine. 2010;363(26):2477-81.

[A3] Goldhahn J, Beaton D, Ladd A, Macdermid J, Hoang-Kim A. Recommendation for measuring clinical outcome in distal radius fractures: a core set of domains for standardized reporting in clinical practice and research. Arch Orthop Trauma Surg. 2014;134(2):197-205.

[A4] Wisco OJ, Imbriano P, Zullo M, Adelson D. Aligning research core outcome set development with clinical care performance measurement. Journal of the American Academy of Dermatology. 2019;81(2):654-5.

[A5] WHO. WHO Constitution. Geneva, Swizerland: WHO; 1948.

[A6] Fries JF, Spitz P, Kraines RG, Holman HR. Measurement of patient outcome in arthritis. Arthritis & Rheumatism. 1980;23(2):137-45.

[A7] Tarlov A, Ware J, Greenfield S, Nelson E, Perrin E, Zubkoff M. The Medical Outcomes Study: An Application of Methods for Monitoring the Results of Medical Care. JAMA : the journal of the American Medical Association. 1989;262:925-30.

[A8] Lang NM, Marek KD. The classification of patient outcomes. Journal of Professional Nursing. 1990;6(3):158-63.

[A9] Kozma CM, Reeder CE, Schulz RM. Economic, clinical, and humanistic outcomes: a planning model for pharmacoeconomic research. Clin Ther. 1993;15(6):1121-32.

[A10] Wilson IB, Cleary PD. Linking Clinical Variables With Health-Related Quality of Life: A Conceptual Model of Patient Outcomes. JAMA: The Journal of the American Medical Association. 1995;273(1):59-65.

[A11] Nelson EC, Mohr JJ, Batalden PB, Plume SK. Improving health care, Part 1: The clinical value compass. The Joint Commission journal on quality improvement. 1996;22(4):243-58.

[A12] Seymour J, Newell D, Shiell A. The quiet revolution: reporting of health outcomes in general medical journals. Australian health review : a publication of the Australian Hospital Association. 1997;20(1):88-99.

[A13] Jennings BM, Staggers N, Brosch LR. A classification scheme for outcome indicators. Journal of Nursing Scholarship. 1999;31(4):381-8.

[A14] Pfeifle WG. Perspectives on clinical outcomes assessment: A view for the allied health professions. Journal of Allied Health. 1999;28(4):240-6.

[A15] Evans DB, Edejer TTT, Lauer J, Frenk J, Murray CJL. Measuring quality: From the system to the provider. International Journal for Quality in Health Care. 2001;13(6):439-46.

[A16] Valderas JM, Alonso J. Patient reported outcome measures: A model-based classification system for research and clinical practice. Quality of Life Research. 2008;17(9):1125-35.

[A17] Wood L, Egger M, Gluud LL, Schulz KF, Jüni P, Altman DG, et al. Empirical evidence of bias in treatment effect estimates in controlled trials with different interventions and outcomes: meta-epidemiological study. BMJ. 2008;336(7644):601.

[A18] Porter M. What Is Value in Health Care? New England Journal of Medicine. 2010;363(26):2477-81.

[A19] Davey J, Turner RM, Clarke MJ, Higgins JP. Characteristics of meta-analyses and their component studies in the Cochrane Database of Systematic Reviews: a cross-sectional, descriptive analysis. BMC Med Res Methodol. 2011;11:160.

[A20] Smith V, Clarke M, Williamson P, Gargon E. Survey of new 2007 and 2011 Cochrane reviews found 37% of prespecified outcomes not reported. J Clin Epidemiol. 2015;68(3):237-45.

[A21] Drummond M. Methods for the economic evaluation of health care programmes. Fourth edition ed. Oxford, United Kingdom ; New York, NY, USA: Oxford University Press; 2015 2015. 445 p.

[A22] WHO. International classification of functioning, disability and health (ICF), English version. Geneva, Swizerland: WHO; 2017.

[A23] Moorhead S, Johnson M, Maas ML, Swanson E. Nursing Outcomes Classification (NOC)-e-book: Measurement of health outcomes. 6th ed: Elsevier Health Sciences; 2018.

[A24] Leavy MB, Schur C, Kassamali FQ, Johnson ME, Sabharwal R, Wallace P, et al. Development of Harmonized Outcome Measures for Use in Patient Registries and Clinical Practice: Methods and Lessons Learned. Rockville (MD): Agency for Healthcare Research and Quality; 2019 2019 Feb. Report No.: 19-EHC008-EF.

[A25] Dodd S, Clarke M, Becker L, Mavergames C, Fish R, Williamson PR. A taxonomy has been developed for outcomes in medical research to help improve knowledge discovery. Journal of Clinical Epidemiology. 2018;96:84-92.

[A26] Boers M, Beaton DE, Shea BJ, Maxwell LJ, Bartlett SJ, Bingham CO, et al. OMERACT Filter 2.1: Elaboration of the Conceptual Framework for Outcome Measurement in Health Intervention Studies. The Journal of Rheumatology. 2019;46(8):1021.

[A27] NIH. Patient-Reported Outcomes Measurement Information System – PROMIS; Accessed 7.7.2020. Available from: <https://www.healthmeasures.net/explore-measurement-systems/promis/intro-to-promis>.

[A28] COMET-initiative. COMET Initiative - Outcome Classification 2019; Accessed 8 October 2020. Available from: <http://www.comet-initiative.org/Resources/OutcomeClassification>.

[A29] Gliklich RE, Leavy MB, Karl J, Campion DM, Levy D, Berliner E. A framework for creating standardized outcome measures for patient registries. Journal of Comparative Effectiveness Research. 2014;3(5):473-80.

[A30] Wisco OJ, Imbriano P, Zullo M, Adelson D. Aligning research core outcome set development with clinical care performance measurement. Journal of the American Academy of Dermatology. 2019;81(2):654-5.
